# Surface decorated quantum dots: Synthesis, properties and role in herbal therapy

**DOI:** 10.3389/fcell.2023.1139671

**Published:** 2023-03-21

**Authors:** Mirza Shahed Baig, Ravikiran Maheshrao Suryawanshi, Mehrukh Zehravi, Hitendra S. Mahajan, Ritesh Rana, Ahemadi Banu, Muthukumar Subramanian, Amit Kumar Kaundal, Sachin Puri, Falak A. Siddiqui, Rohit Sharma, Sharuk L. Khan, Kow-Tong Chen, Talha Bin Emran

**Affiliations:** ^1^ Department of Pharmaceutical Chemistry, Y. B. Chavan College of Pharmacy, Aurangabad, India; ^2^ Department of Pharmaceutics, R. C. Patel Institute of Pharmaceutical Education and Research, Shirpur, Maharashtra, India; ^3^ Department of Clinical Pharmacy Girls Section, Prince Sattam Bin Abdul Aziz University, Al-Kharj, Saudi Arabia; ^4^ Department of Pharmaceutics, Himachal Institute of Pharmaceutical Education and Research (HIPER), Hamirpur, Himachal Pradesh, India; ^5^ Department of Pharmacology, Vishnu Institute of Pharmaceutical Education and Research, Narsapur, India; ^6^ Department of Pharmaceutics, KMCH College of Pharmacy, Coimbatore, Tamil Nadu, India; ^7^ Department of Pharmaceutical Analysis and Quality Assurance, Himachal Institute of Pharmaceutical Education and Research (HIPER), Hamirpur, Himachal Pradesh, India; ^8^ Shobhaben Pratapbhai Patel School of Pharmacy and Technology Management, Mumbai, India; ^9^ Department of Pharmaceutical Chemistry, N.B.S. Institute of Pharmacy, Ausa, Maharashtra, India; ^10^ Department of Rasa Shastra and Bhaishajya Kalpana, Faculty of Ayurveda, Institute of Medical Sciences, Banaras Hindu University, Varanasi, Uttar Pradesh, India; ^11^ Department of Occupational Medicine, Tainan Municipal Hospital, managed by Show Chwan Medical Care Corporation, Tainan, Taiwan; ^12^ Department of Public Health, College of Medicine, National Cheng Kung University, Tainan, Taiwan; ^13^ Department of Pharmacy, BGC Trust University Bangladesh, Chittagong, Bangladesh; ^14^ Department of Pharmacy, Faculty of Allied Health Sciences, Daffodil International University, Dhaka, Bangladesh

**Keywords:** quantum dots, nanoprobe, nanomaterials, biomedical, nanotechnology, herbal medicines

## Abstract

Quantum dots are the serendipitous outcome of materials research. It is the tiny carbonaceous nanoparticles with diameters ranging from 1 to 10 nm. This review is a brief discussion of the synthesis, properties, and biomedical applicability of quantum dots, especially in herbal therapy. As quantum dots are highly polar, they can be surface decorated with several kinds of polar functionalities, such as polymeric molecules, small functional molecules, and so on. The review also consists of the basic physical and optical properties of quantum dots and their excitation―dependent properties in the application section. We focus on therapeutics, where quantum dots are used as drugs or imaging probes. Nanoprobes for several diagnostics are quite new in the biomedical research domain. Quantum dot―based nanoprobes are in high demand due to their excellent fluorescence, non-bleaching nature, biocompatibility, anchoring feasibility for several analytes, and fast point―of―care sensibility. Lastly, we also included a discussion on quantum dot―based drug delivery as phytomedicine.

## 1 Introduction

**FIGURE 1:**
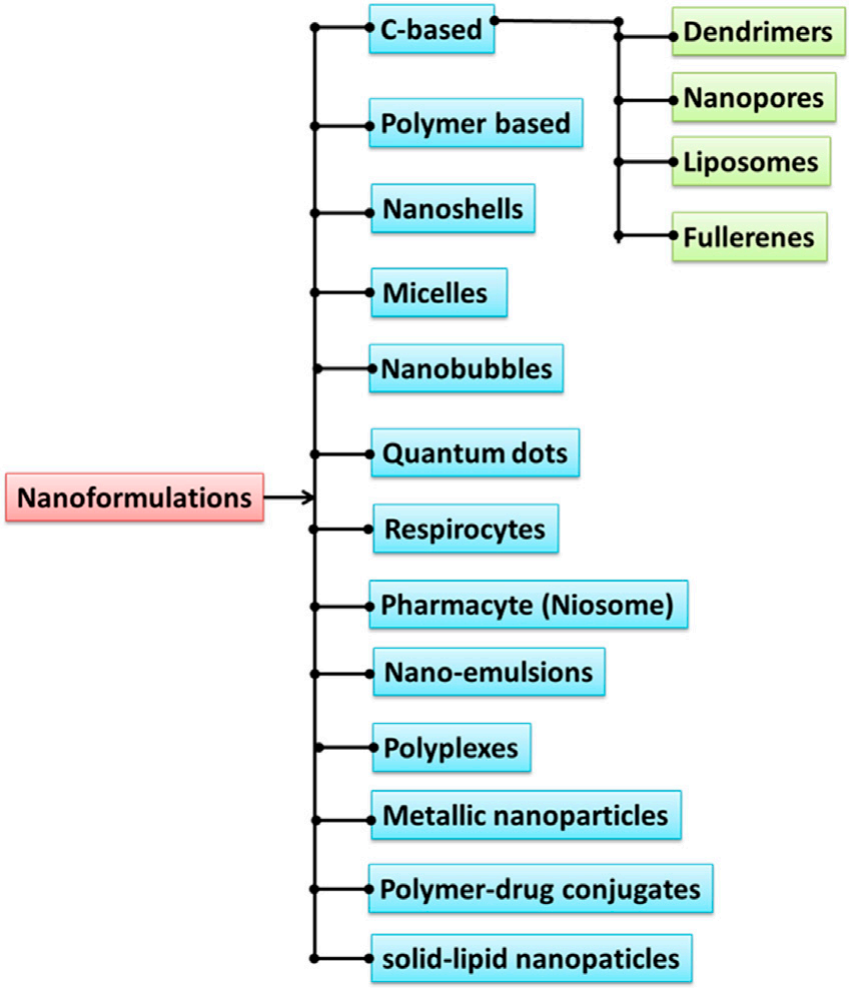
Different types of nanoformulations for therapeutic delivery.

Since ancient times, people all over the world have turned to herbal remedies for treatment, with doctors and patients alike acknowledging the superior therapeutic efficacy of these medications due to their lower risk of side effects (Barkat et al., 2020). To improve patient compliance with phytotherapeutics and decrease the need for recurrent administration, a scientific strategy is required to administer the components in a sustained way. To do this, one must create nanoparticle-based drug delivery systems (NDDS) specifically for the constituents found in herbs. Not only do NDDSs lessen the frequency of dosing needed to overcome non-compliance, but they also help boost the therapeutic value by decreasing toxicity and raising bioavailability. Nanotechnology is one such cutting-edge method. Herbal drug delivery methods on the nanoscale may 1 day help improve plant medicines’ efficacy and curb its drawbacks (Das and Sharangi, 2020). To improve patient compliance with phytotherapeutics and decrease the need for recurrent administration, a scientific strategy is required to administer the components in a sustained way (Kaur et al., 2021). Achieving this goal can be done by creating NDDSs specifically for botanical ingredients. In addition to decreasing the frequency of dosing required to overcome non-compliance, NDDSs also improve the therapeutic efficacy of a drug by decreasing its toxicity, raising its bioavailability, and so on (Sungthongjeen et al., 1999). The discipline of applied science and technology known as nanotechnology focuses on the creation of devices and dosage forms with dimensions between one and one hundred nanometers. Nanomedicine is a term that was used relatively recently to refer to the uses of nanotechnology for the treatment, diagnosis, monitoring, and control of biological systems. The nanocarriers have been created using non-hazardous components, such as synthetic biodegradable polymers, lipids, and polysaccharides, among others. The effectiveness of medicinal herbs is dependent on the overall function of a range of active components. This is because all of the constituents produce synergistic action, which increases the therapeutic value of the herbal medication. Each active ingredient performs an essential function, and those functions are intricately intertwined with one another. However, the vast majority of drugs of herbal origin have an insoluble nature, which results in decreased biodistribution and increased vascular clearance. This necessitates either repeated administration or a higher dose, both of which render the drug an unattractive prospect for use in therapeutic applications. NDDS for phytomedicines incorporates personalized controlled drug delivery, which has shown possibilities to lower the dose frequency, boost the solubility and absorption while decreasing the elimination. Additionally, NDDS for phytomedicines has shown potentials to minimize the amount of time it takes to complete the treatment (Musthaba et al., 2009). When it comes to herbal medicine, the drug delivery systems that are based on nanoparticles (NPs), also known as NDDS, have been recognized to be among the most essential kinds of delivery systems that are available. In addition, the NPs might be particularly used to target the phytomedicines to various organs, cells, and tissues, which would ultimately increase the medicine’s targeting capacity as well as its efficiency and safety. The primary purpose of the design and construction of the NDDS is to circumvent the restrictions that are connected with traditional or conventional herbal medication delivery systems (Musthaba et al., 2009). In the past few years, researchers have paid a lot of attention to the process of creating and testing nanomaterials. Because of their compact size and broad range of applications, these materials have become ubiquitous in virtually every facet of human existence, which has resulted in significant shifts in all of those domains. The uses of nanotechnology include everything from medical instruments to semiconductors and medication delivery systems (Rahimi-Nasrabadi et al., 2016). At some level, every natural or artificial system is made up of nanoscale structures. The formation of useful structures out of individual molecules is one of the goals of nanotechnology. For example, there is a great trend in the formation and characterization of nanoparticles and quantum dots (Hosseinpour-Mashkani and Sobhani-Nasab, 2017). Synthesizing inorganic nanoparticles utilizing natural reducing or capping chemicals such as sugars, vitamins, natural polymers, phytoconstituents, and microbes is an appealing method for achieving this objective. Compounds derived from plants are some of the most promising options for use in the synthesis of nanoparticles and quantum dots on a massive scale. Nanotechnology employs a wide variety of nanoparticles for the administration of drugs, including dendrimers, liposomes, nanopores, and nanoemulsions, amongst others (Figure 1). Herbal components, like those used in traditional Chinese medicine and Ayurveda, have a rich history of use by humans and continue to play significant roles in the maintenance of good health.

A number of factors have attracted researchers in recent years about the nanonization or size reduction of herbal medicines. Increased component solubility, decreased dosage, and enhanced absorbability are only some of the benefits of nanonizing medicines and other bioactive preparations over their crude counterparts (Brigger et al., 2012). Due to their unique properties—larger surface area, electronic structural selectivity between the molecular and metallic states, and processing of a large number of low coordination sites—metallic nanoparticles have found widespread usage in the pharmaceutical and diagnostic industries. In contrast to more traditional methods like centrifugation and filtration, magnetic nanoparticles (MNPs) have been found to be particularly useful in bio-separation, where the conjugation of target biomolecules and MNPs (functionalized with specific receptors) forms complexes that can be easily fascinated in the presence of an applied magnetic field (Kharisov et al., 2014). Bio-sensing, medication administration, MRI, and hyperthermia are just some of the other fields that might benefit from this method (Ganguly et al., 2021). Herbal bioactive or herbal medications have been delivered using solid lipid nanoparticles (SLN) like gold and silver nanoparticles (Möltgen et al., 2013). Drugs or active moieties with anticancer, antibacterial, and anti-inflammatory activity can be delivered by nanogels, which operate as nanocarriers (Kaur et al., 2019). Incorporating the same plant extracts or water-soluble phytoconstituents into the phospholipids to create lipid compatible molecular complexes increases the absorption and bioavailability of the medication or phytoconstituents and is protected by patent (Semalty et al., 2010). Dendrimers, liposomes, nanopores, nanoemulsions, etc. are only few of the nanoparticles used in medication delivery thanks to advancements in nanotechnology. Nanoparticles are an exciting new medication delivery option, but at now, it is impossible to predict how the drug will behave *in vivo*. Typically ranging in size from 2 to 20 nm, quantum dots (QDs) are a semiconducting cluster of atoms that have been chemically created and display intriguing optical features (Parameswaranpillai and Ganguly, 2023). Studies demonstrate that folates adorned nano-formulation selectively targets folate receptor-positive where QDs assisted to decide *in vivo* drug release and drug targeting, which means they may be utilised to target and track certain cancers (Yuan et al., 2014; Das et al., 2022). This article will discuss mostly the synthesis, fabrication, surface functionalization of QDs. Besides these, the article will also discuss the area of QDs in plant derived nanosized drug formulation and their delivery/targeting.

## 2 Quantum dots

**FIGURE 2:**
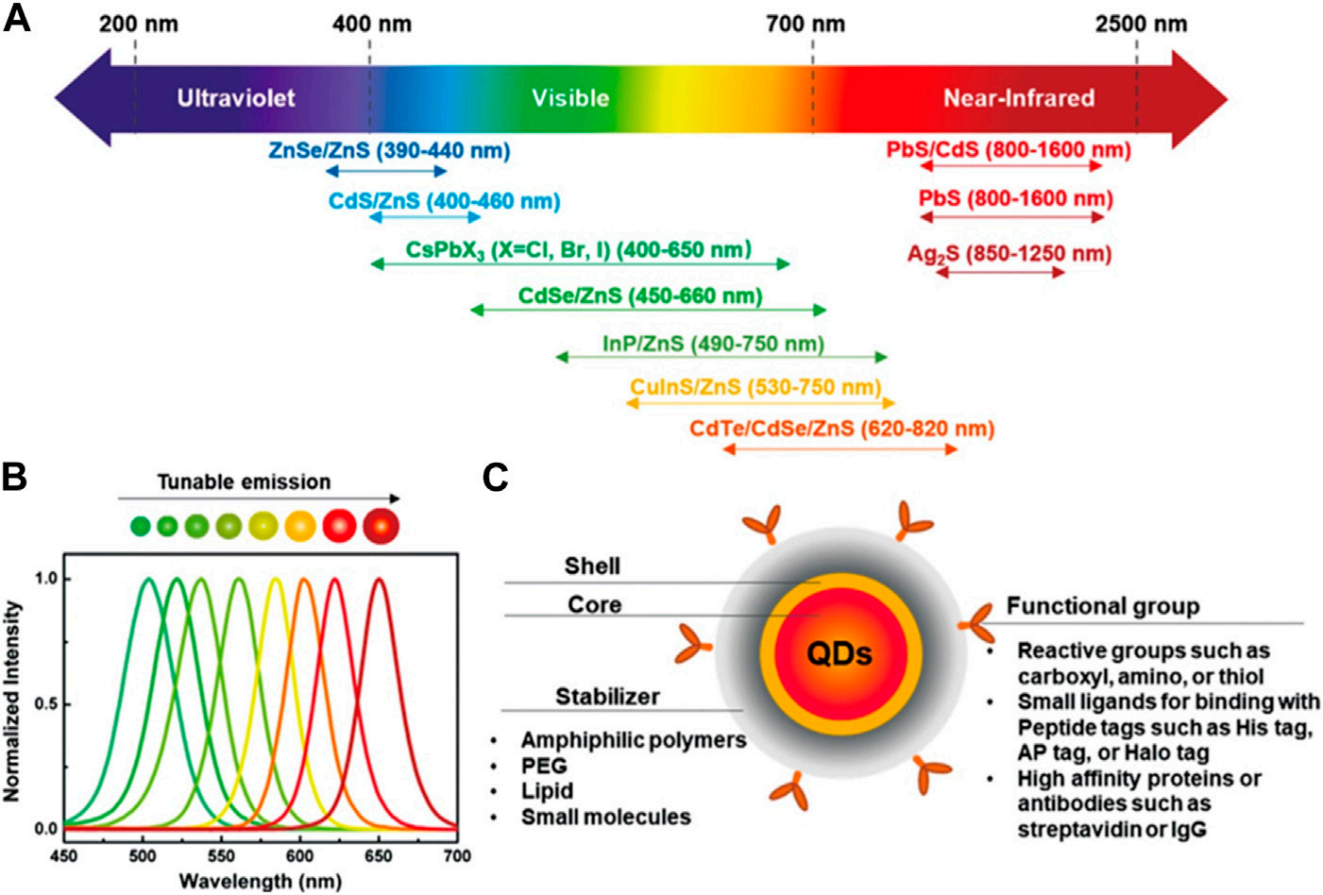
**(A)** Commercially available visible to near infrared emitting QDs. **(B)** Size-controllable emission spectra of CdSe/ZnS QDs. **(C)** A graphical illustration of a QD probe (Wang et al., 2021).

Breakthroughs in the synthesis and manufacturing of novel materials have constantly improved and strengthened the foundation of nanoscience and technology (Ahmed et al., 2014; Lee et al., 2014; Ahmed et al., 2018a). QDs are nanosized semiconductor crystals composed of elements from groups II–VI or III–V. Particles with sizes smaller than the exciton Bohr radius are called QDs (Chan et al., 2002). They are zero-dimensional nanomaterials that only have a limited quantity of electrons, which corresponds to discrete quantized energy in the density of states. Quantum confinement is regarded as the most essential characteristic of QDs (Zorman et al., 1995; Liu et al., 2018b). At low dimensions, the quantum confinement effect predominates, and the properties of the system are distinct from those of bulk materials. Alexei Ekimov, a Russian physicist, made the initial discovery of QDs in solids in the year 1980 (Ekimov et al., 1994). Later on, while working in the Bell laboratory, American chemist Louis E. Brus found them in colloidal solution (Rossetti et al., 1983). The synthesis approaches of QDs can be divided into two categories: top-down and bottom up techniques (Valizadeh et al., 2012). Besides semiconductor QDs, there are several other types of QDs such as carbon quantum dots (Das et al., 2018a; Bhattacharyya et al., 2020; Saravanan et al., 2020; Das et al., 2021a; Das et al., 2021c), silicon QDs (Warner et al., 2005; Chinnathambi et al., 2014), graphitic carbon nitride QDs (Song et al., 2016; Liu et al., 2019a), graphene quantum dots (Bacon et al., 2014; Ahmed et al., 2018b; Das et al., 2019b), magnetic QDs (Koole et al., 2009; Liu et al., 2011; Das et al., 2021b) etc. Due to their wide range of useful features, such as bright multicoloured fluorescence, superior biocompatibility, non-toxicity, good water solubility, and low cost in compared to commercial dyes, these QDs have been receiving a lot of interest (Ahmed et al., 2012a; 2012b; Das et al., 2019a; Das et al., 2019c; Ganguly et al., 2019). QDs have been employed by researchers for different applications in the fields of sensing (Ahmed et al., 2019; Das et al., 2020), cell labelling (Das et al., 2019a), photocatalysis (Ganguly et al., 2019), light-emitting diodes (Zhang et al., 2013), solar cells (Lin et al., 2016; Wang et al., 2016; Gao et al., 2020), batteries and supercapacitors (Bak et al., 2016; Yin et al., 2019; Xu et al., 2021) etc. Figure 2A showed that commercially available QDs are made up of a wide variety of components, and their emission spectra range from the visible to near-infrared spectrum (Wang et al., 2021). The emission spectra of CdSe/ZnS QDs revealed size-tunable properties, as shown in Figure 2B. Pang et al. illustrated the typical structure of QDs (Figure 2C) consists of an inorganic core nanocrystal coated by an organic shell (Wang et al., 2021). The stability of biological buffers can be provided by organic surface coatings such as amphiphilic polymers, phosphoethanolamine (PEG), lipids, or small molecules. Specific binding to biological targets can be achieved through the use of functional groups such as reactive groups, small ligands, streptavidin, and antibodies.

However, as has been discussed in the literature, there are several significant limitations to employing QDs in biological settings (Yao et al., 2014). One of their drawbacks is their toxicity, which results from the release of cadmium, lead, or arsenic from their structure into the biological environment. Their poor solubility is another limitation. Modifying the QDs’ surface has been presented as a current solution to these problems. A few strategies have been proposed in this area, including surface ligand-exchange and covering QDs with biocompatible compounds (e.g., polymer layer). By means of a process known as surface ligand exchange, the naturally hydrophobic ligands on quantum dots (QDs) are swapped out for more hydrophilic ones. Trioctylphosphine oxide (TOPO) and trioctylphosphine (TOP) are two common hydrophobic ligands discovered on the surface of QDs synthesised by the organometallic method, as has been widely described in the literature (Green, 2010). These compounds can increase water solubility and offer adhesion to other molecules like proteins, medicines, or antibodies by binding to the QD surface at one end and anchoring at the other (Karakoti et al., 2015). Luminescence and emission characteristics of QDs’ surfaces as was previously mentioned, the fluorescence characteristics of bare QDs can be suppressed by surface defects formed on crystalline nanoparticles due to their high surface energy (Jamieson et al., 2007). Long-term exposure of QDs to ionic media or cellular media (in biomedical applications) can also cause surface oxidation, photochemical degradation, and leaching of metal ions from the QD core (Singh et al., 2012). To mitigate surface flaws and high reactivity, capping QDs with stable molecules is crucial. Increasing the quantum yield and enhancing the stability of QDs at room temperature are two reasons why ZnS is frequently utilized as a capping agent (Singh et al., 2012). Capable of dissolving in water and other mediums of biological significance—The solubility of QDs in aqueous medium does not increase after capping with an outer shell such as ZnS, despite the fact that QDs are more stable and produce a higher yield. When synthesizing quantum dots (core or core shell), organic solvents (like toluene, octane, and hexane) are typically used, and the dots are stabilized by hydrophobic groups (like amines or phosphines) to regulate their size and prevent further agglomeration at high temperatures. In a single step of synthesis, the core QDs can be coated with a ZnS shell, resulting in cor-shell morphology (Thanh and Green, 2010). However, QDs stabilized with such hydrophobic ligands have limited inherent solubility in aqueous solution. Modifying the surface of QDs with hydrophilic ligands improves their solubility in water.

## 3 General properties of QDs

Researchers are intrigued by QDs due to their exceptional photophysical attributes, including high brightness, remarkable photostability, a broad excitation spectrum, narrow and size-dependent emission, a significant Stokes shift, etc. Scientists have been intrigued by the size-dependent differences in the optical and electrical properties of semiconductor nanocrystals, encouraging them to investigate the possibility of low-dimensional semiconductor materials.

It is feasible to vary the optical band gap, emission energies, and fluorescence features of QDs by adjusting their size as well as their quantum state, or quantum confinement. When the particle size is comparable to the electron’s de Broglie wavelength, the particle nature of electrons transforms to wave nature due to carrier confinement (Shaikh et al., 2021). Quantum mechanical tunnelling takes place between two particles that are close to one another due to the wave nature of electrons. Therefore, semiconductors made up of QDs are able to transport electricity (electrons and holes) in a highly constrained environment while maintaining well-defined energy levels. Their optoelectronic properties change depending on their size and shape. The increase in surface to volume ratio of QDs has led to their widespread application in the production of low-cost, large scale, and flexible thin film optoelectronic devices. As the preparation processes of the QDs are so versatile, there are major differences in the inherent properties of the QDs that are ultimately produced. These differences can be found in the size, shape, or surface arrangements of the QDs, and they ultimately lead to a diversity in the optical properties. Ajayan et al. discovered that the size of the QDs has a significant influence on the band gap, which in turn can result in a variety of different PL emissions depending on the size of the QDs (Peng et al., 2012). By manipulating the temperature, three distinct types of QDs with varied particle size distributions were produced (1–4 nm, 4–8 nm, and 7–11 nm). As the energy gap reduced from 3.90 to 2.80 eV, the PL emission colours of the resultant QDs varied from blue and green to yellow (Baig et al., 2022).

**FIGURE 3:**
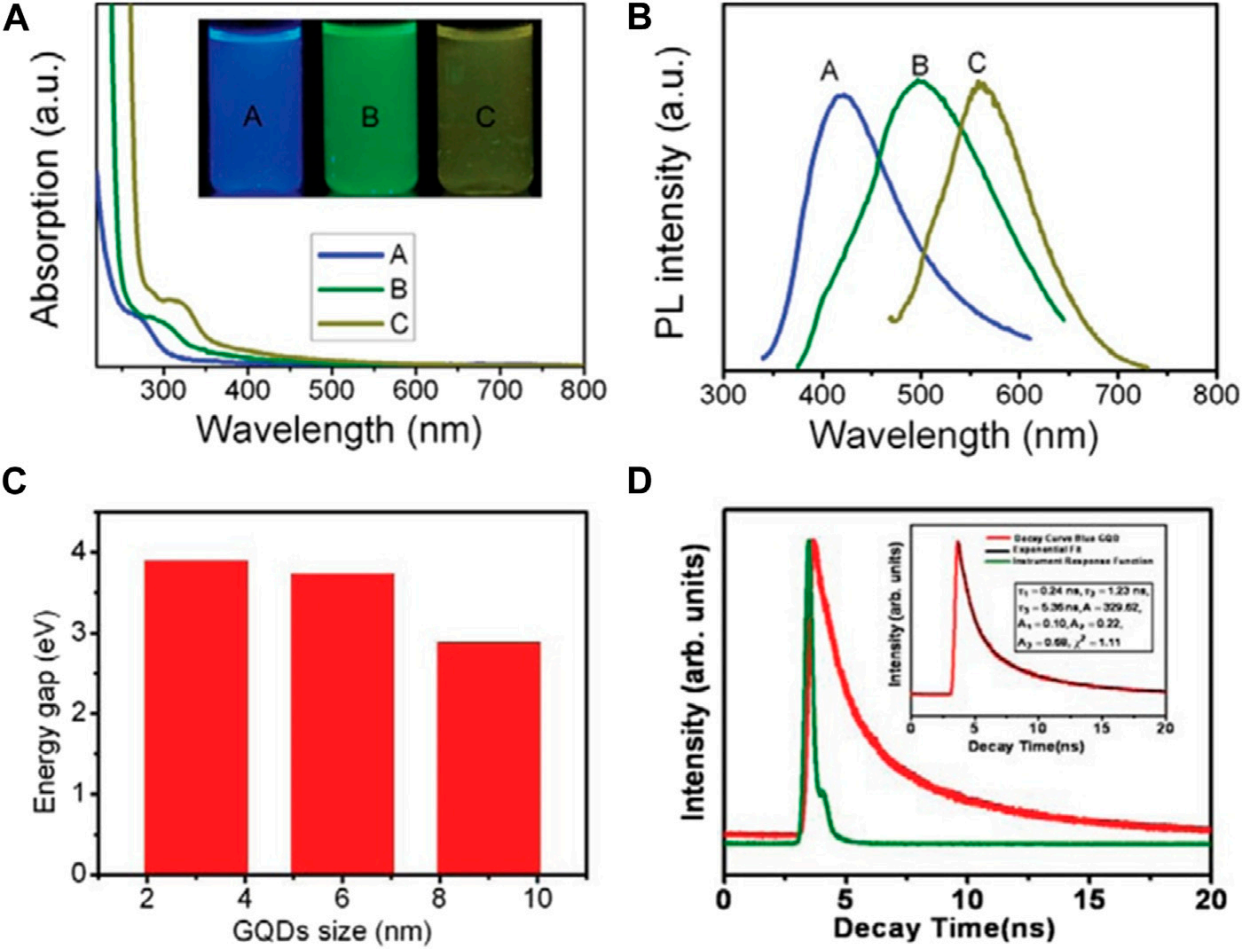
**(A)** UV–Vis absorption spectra of GQDs synthesized at various temperatures A (120°C), B (100°C), and C (80°C), respectively. Inset: the corresponding GQDs under 365 nm UV light irradiation. **(B)** Photoluminescent emission spectra of GQDs with different emission colour excited at 318, 331, and 429 nm, respectively. **(C)** Relationship between the energy gap and the size of GQDs. **(D)** TRPL decay profile of blue GQDs. Reproduced with permission from reference (Peng et al., 2012).

**FIGURE 4:**
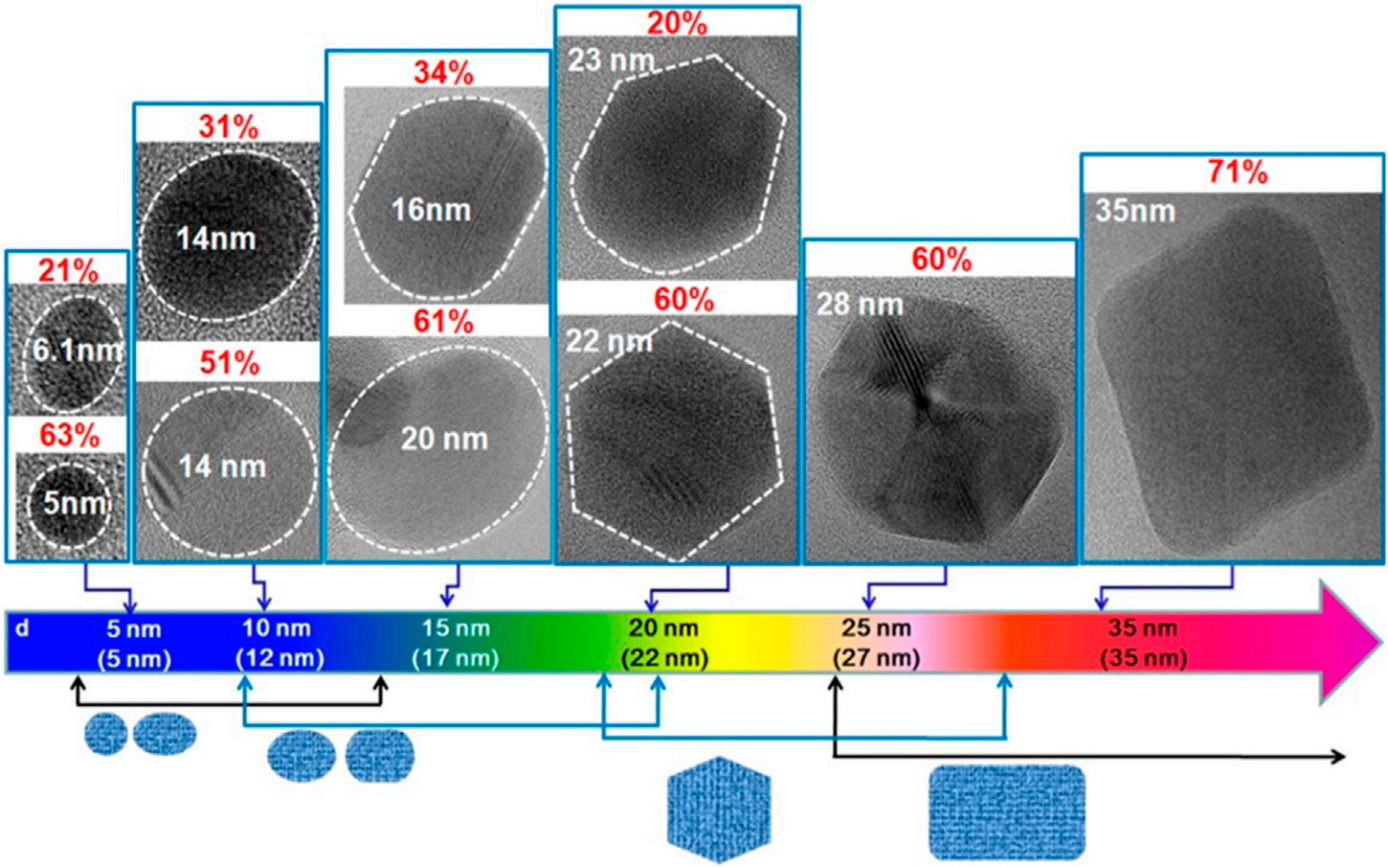
HRTEM images of QDs presented their primary shapes and corresponding populations (p) as the average size of the QDs increases (Kim et al., 2012).

The UV-visible absorption spectra of GQDs produced at three different temperatures (80, 100, and 120°C) were shown in Figure 3A. A distinct blue shift from 330 to 270 nm was detected as the temperature increased. The discovery suggests that the reaction temperature can influence the absorbance properties of as-synthesized GQDs. The inset of Figure 3A showed digital photographs of three different GQDs irradiated with UV radiation. The emission colour of GQDs manufactured at different temperatures varied (blue, green, and yellow). Temperature effects on the emission wavelength distribution of the synthesized GQDs were shown in Figure 3B. Figure 3C depicted the relationship between the energy gap and the size of GQDs. As the size of the GQDs rises, it is evident that the energy gap will decrease, dropping from 3.90 to 2.80 eV. The luminescence decay properties of the blue GQDs were illustrated in Figure 3D. Blue and green GQD lifetime signals were remarkably well-fit to a three-exponential function. Because of their nanosecond lifetime, QDs are interesting candidates for optoelectronic and biomedical research. Kim et al. reported size-dependent shape and edge-state features that influence the absorption and PL characteristics of GQDs (Kim et al., 2012). Below 17 nm, the quantum dots have a propensity to adopt circular or elliptical shape, with zigzag and armchair edges. When the diameters exceeded 17 nm, a polygonal shape with an armchair edge was achieved (Figure 4). The quantum confinement effect has been found to be responsible for the shift in the absorption peak. On the other hand, when the size of the QDs was increased to 17 nm, the photoluminescent spectra displayed non-monotonic patterns. These non-monotonic behaviors can be attributed to the QDs’ size-dependent shape and edge changes.

When considering the practical uses of quantum dots in a variety of domains, the photostability of the QDs is of utmost importance. QDs stability is affected by a number of variables, including temperature, oxygen, and water (Wang et al., 2017; Fu et al., 2018). Continuous exposure of photoexcited QDs in the presence of oxygen molecules causes the production of surface etching, which ultimately leads to the initiation of fluorescence quenching and broadening of the emission spectrum (Pechstedt et al., 2010). Another reason for photobleaching for many QDs is the water sensitivity of organic amino salts. In the presence of oxygen or water molecules, photoexcited QDs generate free radicals. Then, amino salts react with free radicals, resulting in QDs surface defects (You et al., 2014; Chen et al., 2016; Huang et al., 2017). Because most light-emitting devices operate at temperatures above room temperature, the stability of QDs at higher temperatures is significant. The introduction of protective shells to the surface of QDs plays a crucial role in preserving their stability, quantum yields, and preventing photodegradation.

## 4 Background of QDs in herbal nanotechnology

Herbal supplements are frequently used in conjunction with pharmaceutical treatments, which might increase the risk of adverse medication reactions. Clinically significant interactions between herbs and drugs have been recorded, albeit much of this data comes from anecdotes and small samples of patients. Warfarin users who took Salvia miltiorrhiza with their treatment for prolonged periods experienced increased anticoagulation and bleeding, according to published case reports (danshen). Saquinavir’s AUC and maximum plasma concentration were reduced by *Allium sativum* (garlic), but not by ritonavir or paracetamol (acetaminophen). When combined with chlorpropamide, *Allium sativum* enhanced the clotting time and international normalized ratio of warfarin and triggered hypoglycemia. Combining *Ginkgo biloba* (ginkgo) with warfarin or aspirin (acetylsalicylic acid) led to bleeding, whereas combining ginkgo with a thiazide diuretic led to high blood pressure and coma (Watanabe et al., 2001). While *Panax ginseng* (ginseng) boosted the efficiency of influenza vaccination, it decreased blood concentrations of alcohol (ethanol) and warfarin and produced mania when administered concurrently with phenelzine. Cancer patients who used irinotecan experienced less gastrointestinal distress after taking *Scutellaria baicalensis* (huangqin). Patients on levodopa for parkinsonism reported longer “off” times when given kava, also known as Piper methysticum, and a semicomatose condition when given kava along with the tranquillizer alprazolam. While kava did increase alcohol’s sedative effects on mice, this was not the case for people. Human trough levels of indinavir were lowered by the use of *Silybum marianum* (milk thistle) (Hu et al., 2005). The piperine in black pepper (*Piper nigrum Linn*) and long pepper (*P. longum Linn*) raised the AUC of phenytoin, propranolol, and theophylline in healthy volunteers and plasma concentrations of rifamipicin (rifampin) in patients with pulmonary TB.

**FIGURE 5:**
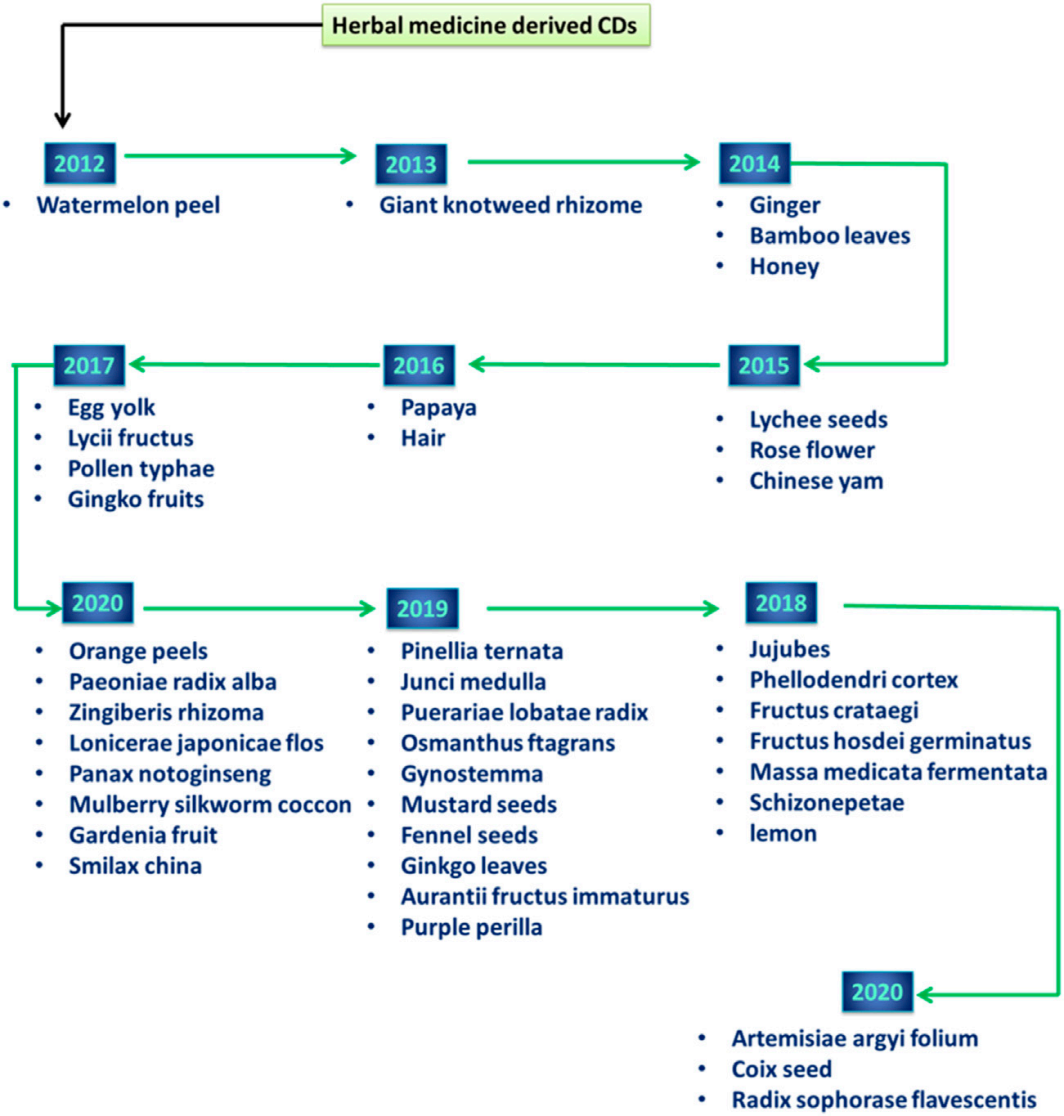
Timeline of herbal medicine derived CDs.

Zhou et al. created a water-soluble fluorescent CD as early as 2012 by utilizing the peel of watermelon, which is both a waste product and a raw material that may be replicated (Zhou et al., 2012). A concise timeline of the QDs based research derived from different natural resources have been depicted in Figure 5. This low-cost and fluorescent nanostructure is appealing not just for the development of future nanosensors but also for large-scale applications in high-performance imaging due to the ease with which it can be synthesized as well as the remarkable functional quality it possesses. The C-dots that were synthesized had good water-solubility, a tiny particle size (less than 2.0 nm), a high luminous efficacy, and have been used well in live cell imaging. As of right now, tens of different herbal medicines have been effectively employed as precursors in the synthesis of various herbal medications in the form of CDs. The first herbal medicine recipe for generating HM-CDs was *Jiaosanxian* (JSX), which was a mix of *Fructus Crataegi* (Jiaoshanzha), *Fructus Hordei Germinatus* (Jiaomaiya), and *Massa Medicata Fermentata* (Jiaohenqu). Following this, JSX-CDs established important benchmarks in the development of CDs generated from herbal medicine formulations. Plants serve not only as the major source of medicinal ingredients but also as the principal therapeutic agents in herbal medicine systems. Because there is no comprehensive report on the components of HM-CDs that are responsible for their activity, we are unable to expound on the possible connection that exists between HM-CDs formed from various medicinal parts and the components that make up their source ingredients. CDs obtained from the same section of several herbal species each have their own unique set of characteristics. The scientists created CDs that were obtained from 14 distinct strains of orange peels, and this study is considered to be the most typical one. Under the identical circumstances of preparation, the QY values produced by the several kinds varied significantly from one another (Wang et al., 2020). This work provides more evidence that the QY may be connected with the quantities of volatile oils, which would drive the investigation of HM-CDs obtained from the pericarp if it were to be successful.

## 5 Bioinspired QD medication delivery

In recent years, there has been a growing interest in nanopharmaceuticals, which has resulted in a number of technological developments with an emphasis on the development of new applications. The active phytoconstituents or standardized extracts of plants are what are used to make nanophytomedicines (Ganguly and Margel, 2021; Ganguly et al., 2022). It was discovered that a nanotized herbal medication that contained the active ingredients of seawort, cassia twig, and liquorice root was successful in treating cancers of the liver, lungs, bones, and skin.

The capacity to identify illnesses at much earlier stages is another benefit offered by nanotechnology (Mitchell et al., 2021). This includes the ability to locate concealed or overt metastatic colonies, which are frequently detected in people who have been diagnosed with breast, lung, colon, testicular, and ovarian cancer (Das et al., 2021b). The concept of theranostic nanomedicine revolves around the integration of diagnostic and therapeutic capabilities into a single drug delivery device.

Theranostics is a relatively new therapeutic paradigm that allows for the simultaneous application of treatment and diagnostics (Khizar et al., 2021). Theranostic medicines provide illness diagnosis, therapy, and real-time monitoring of treatment progress and success, all with one pharmacological agent by integrating both diagnostic and therapeutic capabilities in a single delivery formulation (Das et al., 2019c). Because cancer may take many different forms, there is no single treatment that is certain to be successful for every individual affected by the disease. The ability to monitor drug accumulation in target tissues and therapeutic responses enables an individualized feedback process (Truong et al., 2015). This process allows treatment strategies (drug doses, patient management protocols, and so on) to be further adjusted to meet the changing requirements of each individual patient (Cho et al., 2008). As a result, theranostics is an example of a technology that makes individualized medicine possible. Drug delivery applications systems are typically based on biocompatible and biodegradable polymers that are constructed to frame nanocarriers. These nanostructures have the ability to co-encapsulate or conjugate anti-cancer drugs, contrast agents, and preferential ligands (Li et al., 2017). These nanocomposites are developed to specifically target cells in order to improve diagnosis, evaluation, and resolution of pathologies. It is widely agreed upon that the utilization of nanoscale carriers is required in order to accomplish lengthy blood circulation (by evading both renal clearance and hepatic capture) and significant accumulation in tumour tissue (Hui et al., 2019). As a rule, tumours have leaky vasculature and inefficient lymphatic drainage, both of which contribute to increased nanoparticle penetration and prolonged retention times. The improved permeability and retention effect, often referred as this passive targeting mechanism, has been extensively highlighted as a basis for the use of nanocarriers in cancer treatment.

## 6 QDs-based therapeutics

In recent times, among the natural raw material that may be utilized for the creation of C-dots, food items have been the subject of research (Ganguly et al., 2019). This is because the hydrothermal process that is involved in the synthesis is a straightforward one that is also economical and kind to the environment. Cinnamon, red chili pepper, turmeric, and black pepper are examples of popular spices that have been the subject of extensive research due to the long-standing belief that they possess therapeutic qualities (Das et al., 2018b). For example, piperine, a key chemical ingredient that may be found in black pepper, has been demonstrated to have anti-inflammatory benefits, as well as anti-angiogenic and anti-arthritic effects. Using a one-pot green hydrothermal approach, one piece of research reported the successful synthesis of aqueous fluorescent C-dots from cinnamon, red chili, turmeric, and black pepper (Vasimalai et al., 2018). Various toxicological effects are exhibited by the C-dots in accordance with the particular source that was used for the synthesis. Incubating tumour cells with these C-dots can reduce tumour development. A previously unreported discovery of this fundamental nature provides a promising new avenue for investigating potential medicinal applications.

Repairing the blood-brain barrier (BBB) after traumatic brain injury (TBI) is an ongoing multi-faceted problem. Luo et al. described an unique and non-toxic functional negatively-charged carbon dots (CDs) that were created from green Semen pruni persicae and Carthamus tinctorius L. (TH-CDs) using a hydrothermal biosynthesis without the use of any organic solvent (Luo et al., 2022). Through the injection of TH-CDs into the tail vein of mouse models, it was possible to enhance neurological function, brain edema, neuronal damage, and the permeability of the BBB without causing systemic toxicity. This research showed an affordable, environmentally friendly, non-toxic, and intravenous functioning TH-CD that might be a feasible therapy option for traumatic brain injury (TBI).

A team of researchers found and analyzed the compounds known as Aurantii fructus immaturus carbonisata (AFIC)-CDs, and then studied the bioactivities of these compounds. They evaluated the anti-gouty arthritis and anti-hyperuricemia activities of AFIC-CDs, as well as investigated the probable mechanisms underlying these activities (Wang et al., 2019). This study provided further evidence that the newly discovered AFIC-CDs successfully decreased hyperuricemia by inhibiting the activities of XOD in both blood and hepatic tissue.

The identification of iodide ions has thus far been accomplished by the use of various fluorescence detectors that are based on CDs. The iodine ion is one of the most essential anions found in living creatures. It plays a crucial role in the production of thyroid hormones, which makes it one of the most important anions overall. A number of thyroid illnesses, including goitre, cretinism, hypothyroidism, autoimmune thyroid diseases, and even an increase in the risk of thyroid cancer, have been linked to iodine shortage and iodine excess, respectively (Liu J. et al., 2019). Since the human body is unable to produce iodine on its own, the human body must obtain it from other sources, most often drinking water and a wide variety of foods. Curcumin is a yellow-orange polyphenol chemical that is obtained from the rhizome of the plant Curcuma longa L. Curcumin has been utilized in several applications, including as a flavor and organic colouring agent, as well as in food additives, cosmetics, and traditional Chinese medicine. Furthermore, an excessive amount of curcumin can render DNA inactive, bring about a drop in the amount of ATP found inside cells, and set off the necrosis process in tissues. A multi-mechanism recognition for iodide and curcumin in actual complicated biological and dietary samples was achieved by Tang et al. using nitrogen-doped fluorescent carbon dots (NCDs) (Tang et al., 2021). The NCDs that were synthesized may also be employed as a fluorescence quenched sensor for the detection of curcumin. This sensor is based on the synergistic internal filtering effect (IFE) and static quenching, and it has a reasonable detection limit of 29.8 nM and a good linear range of 0.1–20 μM. According to these findings, carbon dots have the potential to be used as sensing materials for the detection of iodine and curcumin, both of which are beneficial to human health.

Even though it has been demonstrated that ligand fishing is an effective method for the identification of bioactive components from complicated mixtures like those found in natural goods, this method cannot be used to the analysis of biological images. To fish Hsp90 ligands, a Hsp90 coated silica-InP/ZnS QDs nanoparticles were shown (Hu et al., 2018). It is focused on selective separation of ligands by the use of Hsp90 MSN-QDs conjugates, followed by further analysis through HPLC/MS and GC-MS. The method offers a platform that is both quick and reliable for the discovery of compounds with biological activity.

Liu et al. reported their attention on a newly discovered chemical known as Phellodendri Cortex Carbonisatus-carbon dots (PCC-CDs). These carbon dots were found in the PCC aqueous extract but were not present in the crude herb itself. We succeeded in isolating and naming previously unknown PCC-CDs (Liu et al., 2018a). Both the mouse tail amputee and the liver scratched models were utilized in order to investigate the hemostatic impact that PCC-CDs had. In all injury scenarios, the application of PCC-CDs resulted in a considerable reduction in the amount of time spent bleeding.

**FIGURE 6:**
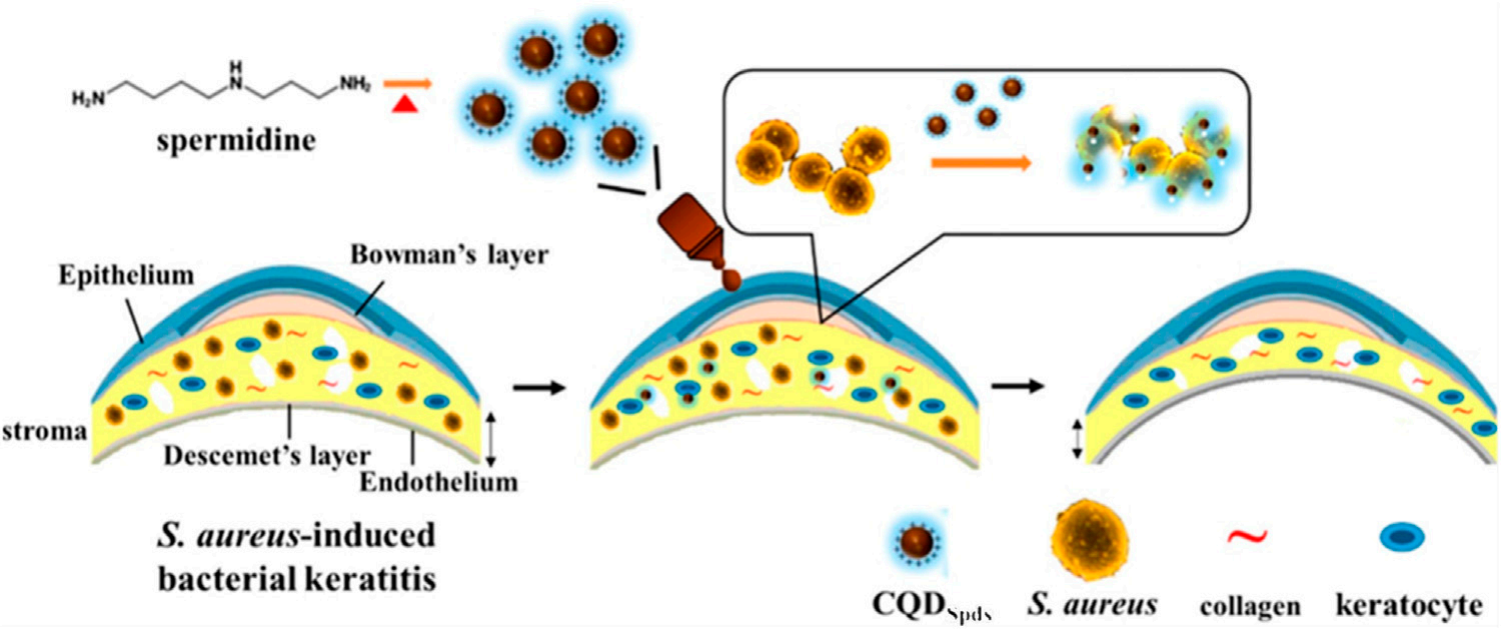
Schematic of one-step dry heating synthesis of CQDSpds from spermidine and their treatment for *S. aureus*-induced bacterial Keratitis (Jian et al., 2017).

Jian et al. came up with a one-step process to synthesis carbon quantum dots (CQDPAs) from biogenic polyamines (PAs) in order to use them as an antimicrobial agent for the external treatment of bacterial keratitis (BK) (Jian et al., 2017). Infectious keratitis is brought on by inflammation of the cornea, which is brought on by microbial infections brought on by bacteria, viruses, fungus, or parasites. These illnesses can cause infectious keratitis. CQDs that are produced by the straight decomposition of spermidine (Spd) powder using a straightforward process including dry heating have a solubility and yield that is significantly greater than those produced from putrescine and spermine (Figure 6). They discovered that the super-cationic property of CQDSpds (ζ-potential of about +45 mV) can stimulate the loosening of the tight connection between ocular epithelial cells. Moreover, the topical administration of CQDSpds as a medicine demonstrates substantially greater efficacy in the treatment of BK compared to Ag NPs. This is because super-cationic CQDSpds-induced relaxation of the vascular endothelium of corneal epithelial cells for its paracellular transport.

**FIGURE 7:**
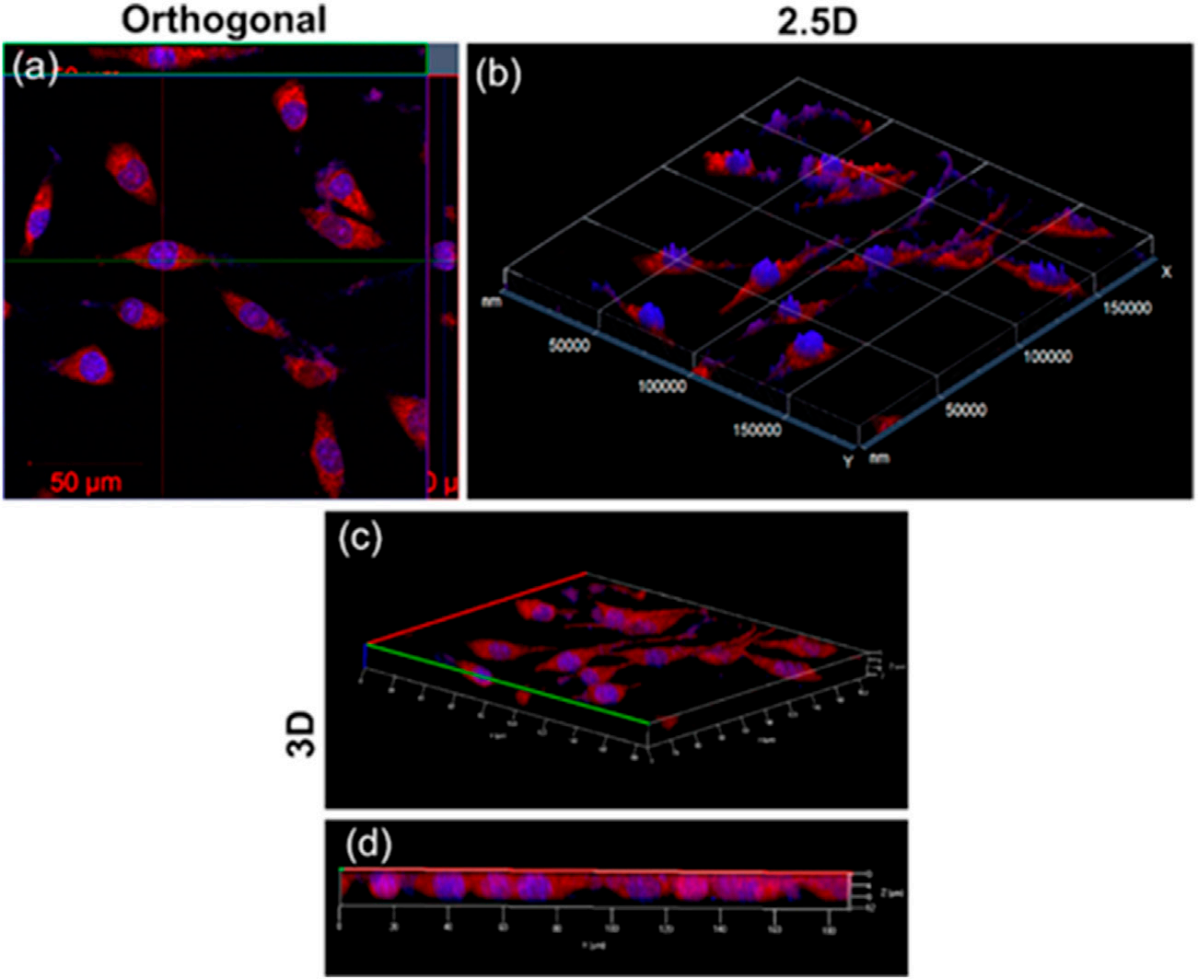
Intracellular mGQDs in DAPI-stained L929 cells. **(A)** Optical slice with *x*-, *y*-axes and projections of *x*-, *z*-and *y*-, *z*-axes of L929 cells; **(B)** 2.5D imaging and **(C,D)** 3D intracellular imaging exhibiting localization signals from DAPI-stained nuclei and mGQD-stained cytoplasm (Kumawat et al., 2017).

**FIGURE 8:**
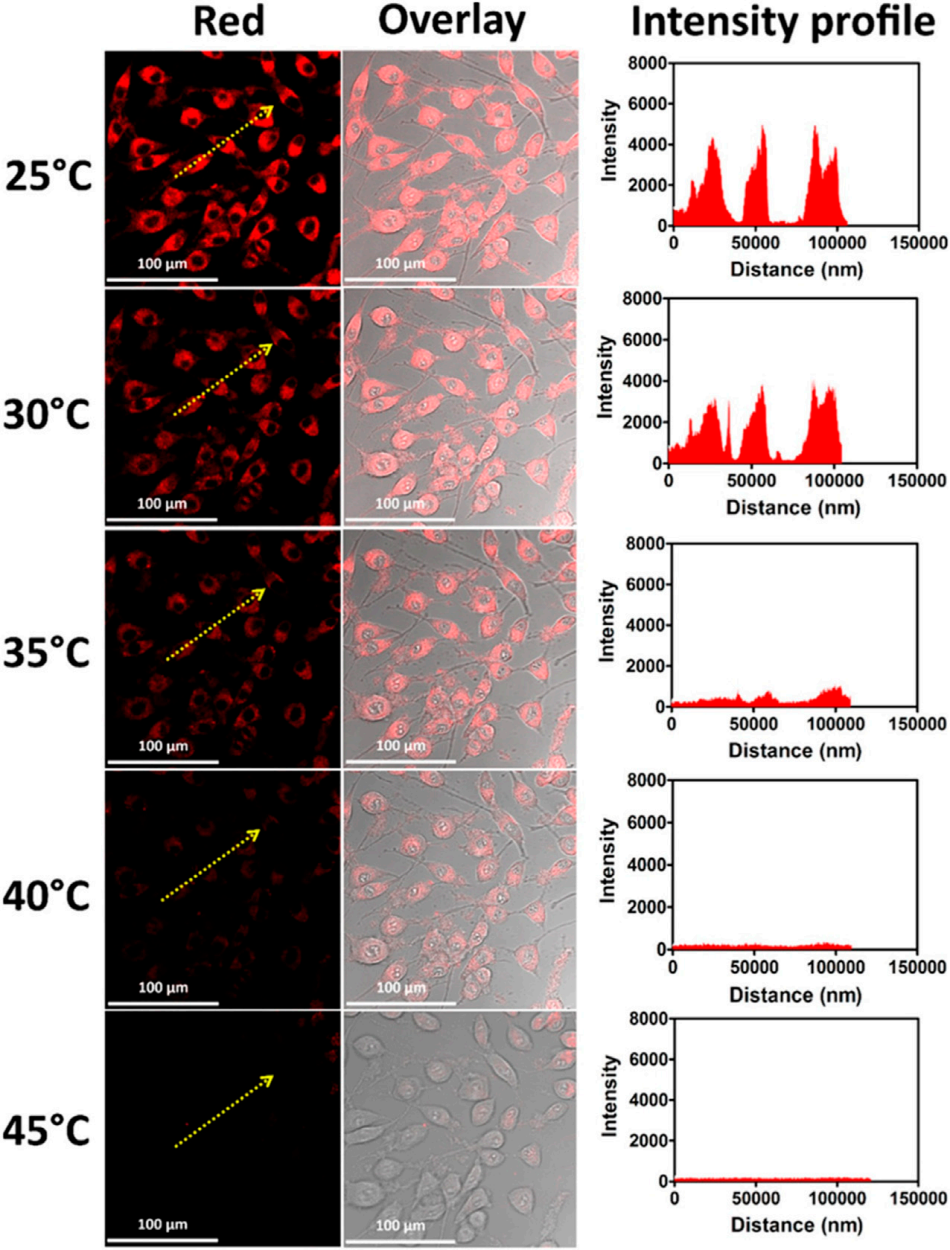
L929 cells with 0.1 mg/mL mGQDs for intracellular temperature sensing. Using CLSM’s temperature controller, a typical area was heated to 25 to 45°C (Kumawat et al., 2017).

*Mangifera indica* (mango) leaf ethanolic extracts were used in a facile one-pot microwave-assisted green synthesis process reported by Kumawat et al. for the creation of brilliant red-luminescent graphene quantum dots (GQDs) (Kumawat et al., 2017). Even at high concentrations (0.1 mg/mL) 24 h after treatment, these mGQDs were shown to have outstanding biocompatibility and demonstrated 100% cellular absorption on L929 cells (Figure 7). Additionally, it was established that the mGQDs may function as NIR-responsive fluorescent bioimaging probes. These probes self-localize themselves specifically within the cytoplasm of the cell. Because of its superior biocompatibility and photostability, mGQDs served as a promising candidate for near-infrared imaging and temperature sensing of living cells *in vitro*. This was achieved by using the mGQDs. According to the findings of research on how changes in temperature affect the fluorescence of tissue, the intensity of the tissue’s fluorescence is greater when the temperature is lower (Figure 8). At a temperature of 25°C, the mGQDs that had been internalized in L929 cells displayed a bright fluorescence. However, this fluorescence steadily diminished when the temperature was increased up to 45°C. The fact that the fluorescence intensity can be reduced by as much as 95% in response to a temperature change of only 20°C suggests that it can be utilized to identify even the most subtle of temperature shifts inside the cellular environment.

Multiple light source procedures, including those involving lasers and LEDs, have undergone continuous development for the purpose of incorporation into medical protocols. These functions are made possible due to the fact that they may cause photochemical reactions to take place on a cellular level. The practice of using light as a medical therapy has gained popularity in recent years due to the fact that it is both non-invasive and non-toxic (Yin et al., 2015). Carbon dots have shown promise as a potential use in the fight against many kinds of corona viruses. In areas of biomedicine other than treatment, such as biosensing, bioimaging, and so on, there has been a significant amount of study on carbon dots. When compared to alternative nanoparticulate therapeutic delivery methods, carbon dots stand out as the most excellent option due to their surface functionalization and minimal toxicity. In the not too distant future, the creation of biocompatible nanotherapeutics for addressing infectious diseases may continue to use these functionalized carbon dots as a new stage in the process.

## 7 Summary and future outlook

The application of nanotechnology in the delivery of herbal medicines has the potential to improve the medicines’ biological activity and contribute to the solution of problems that are often associated with the consumption of herbal medicines. Despite this, there is still a great deal of challenges to be conquered before it will be possible to implement clinically effective treatments in this field. The use of these technologies in therapeutic contexts is now plagued by a number of challenges, one of which is the evaluation of novel approaches to the management of the interactions of nanoparticles with biological beings. In order for nanopharmacology and treatments to be successful, there are a number of challenges that need to be surmounted, some of the most prominent of which are the wide range of created nanomaterials and the extensive number of potential side effects. In a nutshell, the purpose of this review was to highlight the potential variety of QDs, as well as their naturally plentiful resources and their uses in the field of herbal therapy. The first portion covered not only the quantum dots’ optical properties and surface polarities, which are crucial to know in order to put into practice their real mode of action, but also the quantum dots’ physical characteristics, which were covered in the previous section. The globalization of both commerce and the market has made it feasible for many kinds of herbal supplements to be combined into a single product across the world. At the present time, herbal medicines and other products associated with herbal medicine that are available for purchase on markets all over the world are derived from plants that are native to either China, India, the Middle East, or the Western world. It is anticipated that the efficacy and useful applicability of natural goods and herbal therapies that are administered in conjunction with the nanocarrier will result in an increase in the significance of preexisting drug delivery systems. These natural remedies and herbal treatments are given in combination with the nanocarrier.

Herbal medicine as a precursor is receiving a growing amount of attention as a result of the substantial growth in the number of raw material options that are available for the synthesis of QDs. QDs obtained from herbal medicine (also known as HM-QDs) are the most recent member to be added to the family of QDs when compared to other types of biomass precursors. In the last 10 years, a vast number of studies have shown that HM-QDs have a tendency to be effective at theranostics even when drug loading is not performed. QDs have been of crucial significance to the field of bio-nanotechnology throughout the course of the past 10 years. Researchers had envisioned a wide range of uses for QDs, many of which are not achievable with organic fluorophores due to the one-of-a-kind and aesthetically pleasing emission that they produce. In addition to QDs, additional nanostructures, such as metallic and carbon-based nanoparticles, were introduced to the mixture as precursors. These nanostructures have the potential to speed up biological research that is both basic and applied. The incorporation of nanostructures into analytical instruments will be one of the other areas of study that will converge. Multiplexing, or the screening of vast quantities of proteins and genes concurrently, would be an appropriate capability for such devices, along with single-molecule detection (the ultimate level of detection). In addition, multimodal imaging probes and detection systems should both be topics of greater attention. Already, QDs have been incorporated into bimodal imaging probes. These probes combine the light microscopy capabilities of QDs with the magnetic resonance imaging (MRI) and electron microscopy (EM) modalities. There is a wide variety of potential uses for this technology, ranging from the clinical detection of HIV to the screening of agricultural illnesses to the monitoring of pharmacological compounds in living organisms.
